# Exploration of *Vulcanodinium rugosum* Toxins and their Metabolism Products in Mussels from the Ingril Lagoon Hotspot in France

**DOI:** 10.3390/md21080429

**Published:** 2023-07-29

**Authors:** Vincent Hort, Isabel Bastardo-Fernández, Marina Nicolas

**Affiliations:** Laboratory for Food Safety, Pesticides and Marine Biotoxins Unit, French Agency for Food, Environmental and Occupational Health & Safety (Anses), Paris-Est University, 14 Rue Pierre et Marie Curie, F-94701 Maisons-Alfort, France; isabel.bastardofernandez@anses.fr (I.B.-F.); marina.nicolas@anses.fr (M.N.)

**Keywords:** emerging marine biotoxins, pinnatoxin-G fatty acid esters, mass spectrometry, pinnatoxins, portimines, pteriatoxins, *Vulcanodinium rugosum*, shellfish

## Abstract

Over the year 2018, we assessed toxin contamination of shellfish collected on a monthly basis in Ingril Lagoon, France, a site known as a hotspot for *Vulcanodinium rugosum* growth. This short time-series study gave an overview of the presence and seasonal variability of pinnatoxins, pteriatoxins, portimines and kabirimine, all associated with *V. rugosum*, in shellfish. Suspect screening and targeted analysis approaches were implemented by means of liquid chromatography coupled to both low- and high-resolution mass spectrometry. We detected pinnatoxin-A and pinnatoxin-G throughout the year, with maximum levels for each one observed in June (6.7 µg/kg for pinnatoxin-A; 467.5 µg/kg for pinnatoxin-G), whereas portimine-A was detected between May and September (maximum level = 75.6 µg/kg). One of the main findings was the identification of a series of fatty acid esters of pinnatoxin-G (*n* = 13) although the levels detected were low. The profile was dominated by the palmitic acid conjugation of pinnatoxin-G. The other 12 fatty acid esters had not been reported in European shellfish to date. In addition, after thorough investigations, two compounds were detected, with one being probably identified as portimine-B, and the other one putatively attributed to pteriatoxins. If available, reference materials would have ensured full identification. Monitoring of these *V. rugosum* emerging toxins and their biotransformation products will contribute towards filling the data gaps pointed out in risk assessments and in particular the need for more contamination data for shellfish.

## 1. Introduction

**Figure 1:**
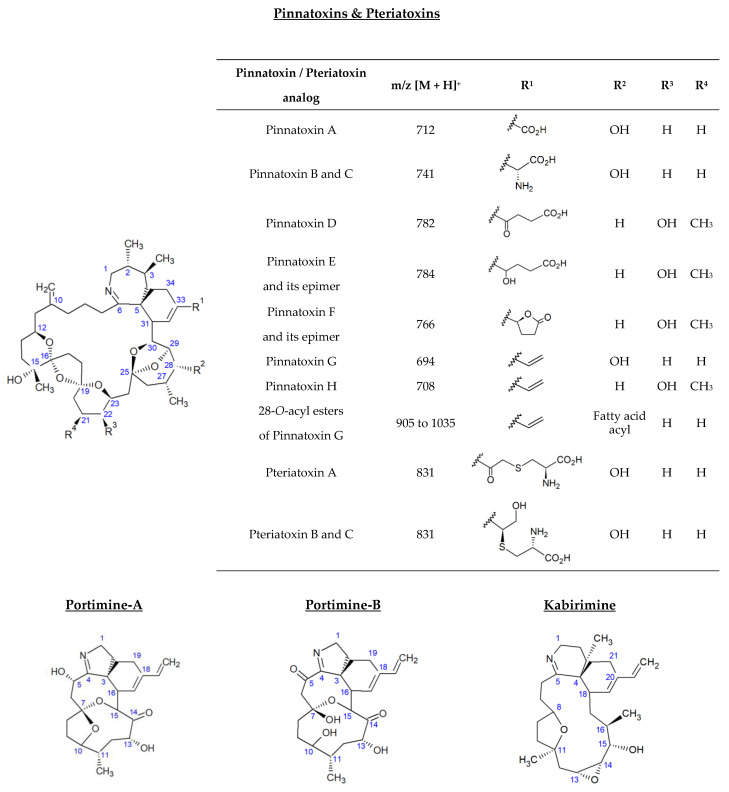
Structure of *V. rugosum* toxins and their metabolites reported in shellfish.

*Vulcanodinium rugosum* is a benthic dinoflagellate first identified in 2010 from water samples collected from Ingril Lagoon on France’s Mediterranean coast [1]. This organism produces a series of cyclic imines considered to be emerging marine biotoxins. Among them, pinnatoxins (PnTXs) were the first identified. Their name is related to the bivalve mollusk species *Pinna attenuata* and *Pinna muricata*, collected in China and Japan, which allowed for the isolation and structural elucidation of the first analogs: PnTX-A, PnTX-B, PnTX-C and PnTX-D [2,3,4,5,6,7,8,9,10]. Isolation of the pinnatoxin-producing dinoflagellates in 2010 subsequently made it possible to identify four additional analogs: PnTX-E, PnTX-F, PnTX-G and PnTX-H [11,12,13,14,15,16]. PnTX-A was also detected from *V. rugosum* isolates, unlike PnTX-B, PnTX-C and PnTX-D. These three analogs are thought to result from shellfish metabolism. As with a range of other phycotoxins, PnTX-G has shown its ability to form a range of 28-*O*-acyl esters after undergoing biotransformation in shellfish. In all, 26 metabolism products have been identified to date [17]. Pteriatoxins (PtTXs), such as pinnatoxins, are polyether macrocycles composed of 6,7-spiro, 5,6-bicyclo and 6,5,6-trispiro ketal rings (Figure 1). Three analogs were isolated from the Japanese bivalve mollusk *Pteria penguin* (PtTX-A, PtTX-B and PtTX-C). None of them have been identified from *V. rugosum* isolates so far, and they are thought to result from the shellfish metabolism of pinnatoxins [11]. In 2013, a new polycyclic ether toxin, named portimine (or portimine-A), was isolated from cultures of *V. rugosum* collected in New Zealand, and its structure was elucidated [18]. The cyclic imine moiety consists of an unprecedented five-membered ring unlike PnTXs and PtTXs, which all contain a six-membered cyclic imine ring. The only structural similarity between these *V. rugosum* metabolites is the presence of a spiro-link to a cyclohexene ring (Figure 1). The bioaccumulation of portimine-A in mussels was first demonstrated in 2020, from samples collected in Ingril Lagoon [19]. In 2019, portimine-B, a new analog of portimine, in which the five-membered cyclic ether is open, was discovered [20]. The same year, a new compound with a cyclic imine was isolated from *V. rugosum* extracts. This metabolite was called kabirimine. Its absolute stereochemistry was elucidated by means of spectroscopic analysis and computational study [21].

The toxicity of *V. rugosum* metabolites has been investigated through different toxicological studies. For PnTXs, depending on different analogs and routes of administration, LD_50_ values established from mouse bioassays vary. Orally, the toxicity of the different analogs can be ranked as follows: PnTX F > PnTX G ~ PnTX H ≫ PnTX E, whereas by the intraperitoneal route: PnTX F > PnTX G~PnTX E > PnTX H > PnTX A [11,12,22,23,24]. For PtTXs, toxicity data are limited. LD_99_ values reported in the literature suggest significant acute toxicity against mice [7]. An in vivo study demonstrated the ability of PnTX-G to cross physiological barriers to reach its molecular target, in particular the intestinal, blood–brain and placental barriers [25]. Interestingly, a recent study including a panel of six cancer cell lines demonstrated that PnTX-G can decrease cancer cell viability, with both cytostatic and cytotoxic effects, opening the way for its use in anticancer therapy [26].

Recently the mode of action of the 28-*O*-palmitoyl ester of PnTX-G was studied by a receptor binding-assay and by two-electrode voltage clamp electrophysiology [19]. An antagonistic behavior of this product of shellfish metabolism towards a nicotinic acetylcholine receptor of the muscle type was demonstrated, with a considerable decrease in antagonistic potency when compared to its precursor neurotoxin. However, the in vivo toxicity of this metabolite cannot be underestimated, as marine biotoxin fatty acid esters can be hydrolyzed by lipases and other enzymes to release free-form toxins into the gastrointestinal tract during human digestion. For instance, the 7-*O*-acyl derivatives of okadaic acid and dinophysistoxin-1 and -2, called dinophysistoxine-3, can undergo hydrolysis to release okadaic acid, dinophysistoxin-1 and/or dinophysistoxin-2 [27,28,29,30]. 

The acute toxicity of portimine-A to mice by intraperitoneal administration (LD_50_ = 1570 µg/kg bw) was much lower compared to many other cyclic imine shellfish toxins. Nevertheless, in vitro assays revealed high cytotoxicity of portimine-A for various cell lines (P388 mammalian cells, Jurkat T-lymphoma cells, mouse embryonic fibroblasts cells and human epidermoid carcinoma cells) and apoptotic activity [18,21,31]. Another in vitro assay using human oral cavity squamous cell carcinoma (OCSCC) cell lines was applied to portimine-A and portimine-B. The latter was less potent than portimine-A but could still induce apoptosis, fragment genomic DNA and reduce cancer cell proliferation in the range of 100−200 nM [20].

It is worth noting that no human cases of poisoning through seafood consumption have been attributed to PnTXs, PtTXs, portimines or kabirimine so far [32]. However, recent studies indicated a close link between *V. rugosum* blooms and acute dermatitis outbreaks involving 60 swimmers in Cuba and artisanal anglers in Senegal. Among the toxins produced by *V. rugosum*, portimine-A and PnTXs were predominant [33,34]. The causal link between these poisoning episodes through direct exposure in seawater and algal blooms still has to be demonstrated.

For *V. rugosum* metabolites, limited occurrence data are available for foodstuffs, and all shellfish focused on. In New Zealand, the toxinic profiles in Pacific oysters (*Crassostrea gigas*) and gastropods (*Cominella glandiformis, Zeacumantus lutulentus, Bursatella leachii*) were dominated by PnTX-E and PnTX-F, with PnTX-D detected to a lesser extent [35,36]. In Australia, PnTX-A, PnTX-D, PnTX-E, PnTX-F and PnTX-G were quantified in Pacific oysters (*Crassostrea gigas*) and razor fish (*Pinna bicolor*) collected in Franklin Harbour, South Australia [11]. In Japan, PnTX-A, PnTX-B, PnTX-C and PnTX-D were identified from Okinawan bivalve *Pinna muricata* [3,4,5,8]. In Canada, Chile, Mozambique and several European countries, PnTX-G was predominant, sometimes with low levels of PnTX-A [17,37,38,39,40,41,42,43,44,45,46]. To date, the highest level quantified in the world was reported in mussels collected in 2009 in Ingril Lagoon, France [39]. A value of 1244 μg PnTX-G/kg was measured. Several samples from the Mediterranean Sea and the Atlantic Ocean also revealed the presence of PnTX-G, but at lower levels. PtTXs were only detected in the bivalve mollusk *Pteria penguin* from Okinawa (Japan) and were never reported elsewhere [7,32]. Portimine-A can be produced in large quantities by Mediterranean strains of *V. rugosum* (IFREMER). Although this toxin seems to have a lower accumulation capacity in shellfish compared to PnTXs, portimine-A was detected in mussels [19]. Portimine-B and kabirimine have never been reported in shellfish or any other marine organisms. 

These metabolites are mainly analyzed by liquid chromatography coupled to tandem mass spectrometry [22,32,47]. For PnTXs and PtTXs, functional assays based on their mechanism of action against nicotinic acetylcholine receptors can also be applied. None of these methods have yet undergone inter-laboratory validation or standardization. The lack of reference material limits and complicates the detection and quantification of these metabolites. To date, only PnTX-A, PnTX-E, PnTX-F, PnTX-G and PnTX-H reference materials are commercially available. 

Risk assessments conducted by EFSA and the French Agency for Food, Environmental and Occupational Health and Safety (Anses) pointed out the need for research to obtain more data on shellfish contamination, and to more accurately estimate the exposure of shellfish consumers to PnTXs [22,32]. PtTXs, portimines and kabirimine were not taken into account in these expert appraisals due to the lack of data available in the literature or their recent discovery. For PnTXs, Anses established that a concentration lower than 23 μg PnTX-G/kg shellfish meat is not expected to result in adverse effects in humans [22].

The aim of this study was to explore the contamination of shellfish collected in Ingril Lagoon, France, in 2018, by *V. rugosum* metabolites and their biotransformation products reported in the literature so far. This well-known hotspot for *V. rugosum* growth is a pertinent place to try to identify its metabolites, and in particular those never reported in Europe, and rarely sought due to the lack of reference materials. Pinnatoxins, pteriatoxins, portimines and kabirimine were screened. We took advantage of the complementarity of liquid chromatography coupled to both low- and high-resolution mass analyzers to monitor shellfish contamination throughout 2018. Liquid chromatography coupled to tandem low- and high-resolution mass spectrometry (LC-MS/HRMS) allowed for qualitative screening, with a high selectivity level, of all the toxins produced by *V. rugosum* and their metabolites, whereas liquid chromatography coupled to tandem low-resolution mass spectrometry (LC-MS/MS) analysis enabled sensitive detection and quantification of toxins in shellfish. When reference materials were lacking, and MS^2^ information was piecemeal or unavailable, thorough investigations enabled obtaining the maximum information about the contamination of shellfish by *V. rugosum* emerging toxins and their metabolism products. This study aimed to address the need for contamination data for *V. rugosum* toxins and their metabolism products, highlighted by risk assessors.

## 2. Results

### 2.1. Pinnatoxins

#### 2.1.1. LC-HRMS Screening of Pinnatoxin Analogs (Instrumental Method A.1)

PnTX-G was identified in all samples collected from Ingril Lagoon at a retention time of 7.1 min. The mass error of the [M + H]^+^ protonated molecule was systematically ≤±5 ppm, and the isotopic pattern of, at least, the M, M+1, M+2 and M+3 isotopes matched with the theoretical profile. In addition to the protonated molecule at m/z 694.46674, the following characteristic product ions of PnTX-G were observed in all the MS^2^ spectra: 164.14322, 440.31573, 458.32574, 658.44629 [M + H − 2H_2_O]^+^ and 676.45648 [M + H − H_2_O]^+^. No fatty acid esters of PnTX-G were detected by HRMS in any sample.

For PnTX-A, a peak at a retention time similar to the standard (6.4 min) was detected in all samples, with a mass error ≤ ± 5 ppm. For samples 18 BM 012, 18 BM 026, 18 BM 122, 18 BM 150, 18 BM 161, 18 BM 194, 18 BM 257, 18 BM 271 and 18 BM 286, the isotopic pattern of, at least, the M, M+1 and M+2 isotopes matched with the theoretical profile. However, no MS^2^ spectrum was triggered though dd-MS^2^ acquisition in any sample, due to the low levels detected.

PnTX-B, PnTX-C, PnTX-D, PnTX-E, PnTX-E methyl ester, PnTX-F and PnTX-H were not detected in any mussel sample.

#### 2.1.2. LC-MS/MS Detection and Quantification of Pinnatoxins (Instrument Method B.1)

PnTXs analogs reported to date were detected and quantified through LC-MS/MS analysis with instrumental method B.1. Only fatty acid esters of PnTX-G were screened with another method (instrumental method B.2) due to the numerous analogs to detect. Results are presented in the following section. Analysis with instrumental method B.1 revealed the presence of free PnTX-A and PnTX-G in all samples (Table 1). In addition to the raw extraction, hydrolysis treatment was performed to indirectly assess the potential presence of PnTX-G ester forms in the shellfish (Total PnTX-G = Free PnTX-G + Hydrolyzed fatty acid ester of PnTX-G). Of note, PnTX-G resists base hydrolysis unlike PnTX-G fatty acid esters, which in turn form PnTX-G [11]. Therefore, the approach implemented was based on the paired difference between the free and total toxin content of shellfish as a first step to assess the potential presence of acetylated forms of PnTX-G, and to select the most useful samples for more in-depth investigations. At the same time, we also processed the data for the other toxins quantified from the same hydrolyzed extracts, even though these conjugated forms have not been reported so far. 

PnTX-G was quantified in all collected samples, with an increase in summer and a decrease in autumn. Maximum values were observed in July, with 467.5 µg/kg free toxin, and 475.2 µg/kg total toxin. The levels measured for free toxins were very close to the levels obtained for total PnTX-G. Considering expanded uncertainty (k = 2), estimated from the expected reproducibility assessed by the Horwitz function revised by Thompson [48], uncertainties systematically overlapped, making it impossible to state whether or not PnTX-G esters are present. Uncertainties ranged between 36 and 44%, depending on the PnTX-G level. Nevertheless, if samples contained esters, they would be at trace levels. 

Low levels of PnTX-A were quantified throughout 2018, with a maximum level of 6.7 µg/kg observed in July, such as for PnTX-G. No peaks were observed for the MS traces corresponding to PnTX-B/C, PnTX-D, PnTX-E or PnTX-F. Consequently, in the absence of standards, these toxins were considered not detected.

#### 2.1.3. LC-MS/MS Identification of Fatty Acid Esters of PnTX-G (Instrumental Method B.2)

LC-MS/HRMS analysis did not enable us to identify any fatty acid esters of PnTX-G. Moreover, levels of free PnTX-G, obtained by LC-MS/MS with instrumental method B.1, were very close to levels measured for total toxin. Considering method uncertainty, it was not possible to conclude whether PnTX-G esters were present in the samples. Therefore, we decided to specifically seek the individual esters of PnTX-G with a sensitive LC-MS/MS method dedicated to the direct detection of fatty acid esters of PnTX-G (instrumental method B.2). 

**Figure 2:**
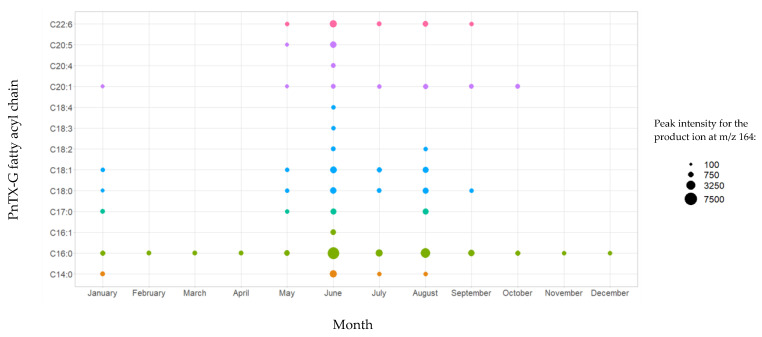
Identification of PnTX-G fatty acid esters by LC-MS/MS in the mussel samples collected from Ingril Lagoon, France, throughout 2018. Graph created with R version 4.0.3, and with the ggplot2 package version 3.3.5 [49,50].

The analysis of non-hydrolyzed extracts revealed the presence of a series of fatty acid esters of PnTX-G, with saturated and unsaturated fatty acid chains ranging from C14 to C22 (Figure 2). These 13 conjugated forms were not observed after hydrolysis treatment of the paired extracts. The retention time of PnTX-G (5.2 min) was lower than that of the acylated compounds (10.0 to 13.7 min), which is consistent with the more lipophilic nature of fatty acid conjugates. For each ester, peaks were observed at the same retention time for the two specific transitions selected, with a signal-to-noise ratio higher than or equal to three. Furthermore, 28-*O*-palmitoyl PnTX-G (C16:0 PnTX-G), the ester with the highest peak intensity, was detected throughout 2018. However, the intensity of the peak was very low compared to PnTX-G. As an example, the peak intensity of C16:0-PnTX-G in the June sample for selected reaction monitoring (SRM) giving the product ion m/z 164 only represented 0.7% of the intensity of PnTX-G. The other esters were detected, with low peak intensities in January and between May and October with different profiles depending on the ester. It is worth noting that the sample collected in June (18 BM 122) contained all the PnTX-G fatty acid esters identified in 2018, and the peak intensities were the highest over 2018 for each of the esters (Figure S1). Although the highest total PnTX-G levels were not observed in June, the period between May and July corresponded to the main upward phase of PnTX-G levels in mussels (Table 1). In contrast with PnTX-G, and similar to the results reported in mussels from eastern Canada by McCarron et al. [17], no fatty acid esters of PnTX-A were detected.

**Figure 3:**
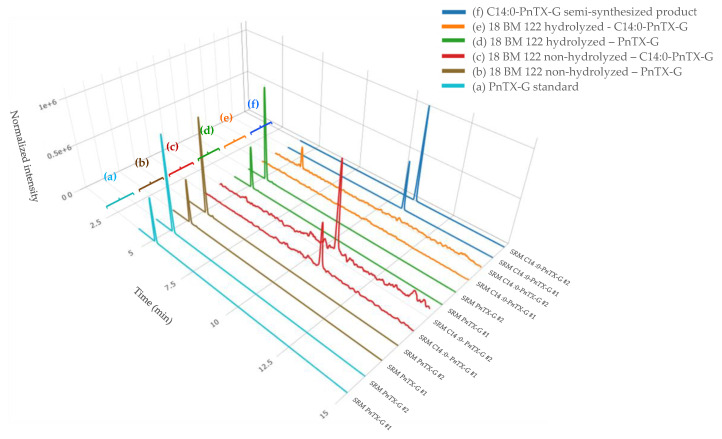
LC-MS/MS identification of PnTX-G, and the C14:0 PnTX-G ester from non-hydrolyzed and hydrolyzed extracts of sample 18 BM 122 (b, c, d, e), and confirmation of its identity after comparison with the standard of PnTX-G (a) and the semi-synthesized products of C14:0 PnTX-G ester (f). SRM #1 and #2 correspond, respectively, to the qualifier and the quantitative transitions of PnTX-G and the C14:0 PnTX-G ester. Graph created with R version 4.0.3, and with the Plotly package version 4.10.0 [49,51].

In a previous study, the discovery of PnTX-G esters was confirmed thanks to the successful production of a C16:0-PnTX-G standard from palmitic anhydride [17]. Based on these results, we decided to also semi-synthesize an ester of PnTX-G to confirm the identification of esters in our mussel samples. Furthermore, 28-*O*-myristoyl PnTX-G (C14:0-PnTX-G) was retained because, to our knowledge, this ester has never been semi-synthesized and because it was the one with the second highest intensity observed in the samples (after C16:0-PnTX-G). We decided to implement the same acylation route as McCarron et al. [17]. The ester was analyzed with instrumental method B.2. The results obtained with this standard and the mussel sample extracts were compared. The retention times of the C14:0-PnTX-G standard and the mussel samples matched perfectly. For sample 18 BM 122, the retention time of the compound suspected to be C14:0-PnTX-G and the standard were the same (10.78 min) (Figure 3). Their respective m/z 458/164 ion ratios were also in agreement. For sample 18 BM 122, this ion ratio was 49.8%, and for the standard it was 52.7%. The relative deviation of both ion ratios (5.5%) leaves no doubt about the identity of the compound detected in mussel samples: C14:0-PnTX-G.

### 2.2. Pteriatoxins

**Figure 4:**
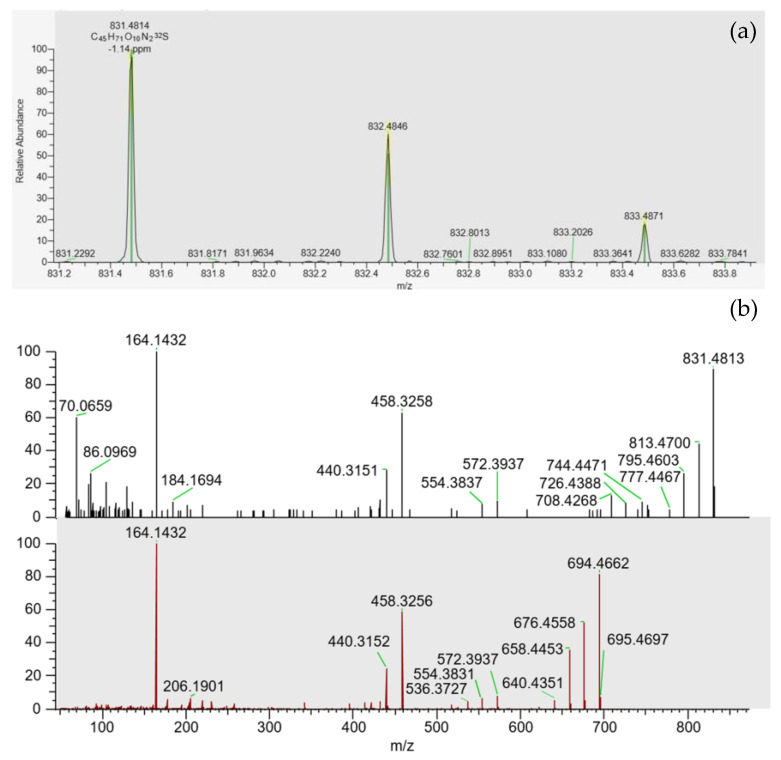
(**a**) Isotopic pattern obtained through tSIM/dd-MS^2^ acquisition, for the peak observed at 5.8 ± 0.1 min in the SPE concentrated extract of the sample collected in August (18 BM 161). (**b**) MS^2^ spectra obtained through PRM acquisition for this compound (top spectrum), and for pinnatoxin-G (bottom spectrum) in this same sample.

PtTX-A, PtTX-B and PtTX-C were also screened in mussels even though no reference materials were available for PtTXs. A peak at a retention time of 5.8 ± 0.1 min was detected through full scan acquisitions in all the samples, except for the mussels collected in December (18 BM 286). The m/z ratio of the precursor ion acquired in full scan was systematically in agreement with the elemental composition of protonated PtTXs [M + H]^+^ (C_45_H_71_O_10_N_2_S; m/z 831.482945) since mass errors were systematically ≤±5 ppm. Additionally, the isotopic pattern of the M, M+1 and M+2 isotopes matched with the theoretical profile in terms of intensity (<25%) and mass error (≤10 ppm). As for PnTX-A and PnTX-G, no [M + NH_4_]^+^, [M + Na]^+^ or [M + K]^+^ adducts were detected. The higher peak intensity was observed in the sample collected in August (18 BM 161). Unfortunately, due to the low levels observed, no spectra were triggered in full MS with data-dependent MS^2^ acquisition mode (full MS/ddMS^2^). Therefore, we decided to concentrate the extract of August twenty times by solid phase extraction (SPE) cleanup to overcome this sensitivity issue, and acquisitions through targeted-selected ion monitoring with data-dependent MS^2^ scans (t-SIM/dd-MS^2^), and parallel reaction monitoring (PRM) were carried out (instrumental method A.2 and A.3). These additional experiments were successful since a rich high-resolution MS^2^ spectrum was obtained (Figure 4). In addition to the precursor ion, we observed product ions at m/z 813.4700 [M + H − H_2_O]^+^, 795.4603 [M + H − 2H_2_O]^+^, 777.4467 [M + H − 3H_2_O]^+^ corresponding to m/z ratios of the three protonated PtTXs which lose one to three water molecules. Takada et al. proposed fragmentation patterns for PtTX-A, PtTX-B and PtTX-C [7]. Three of the product ions indicated by the authors were observed in the present study at m/z 572.3937, 458.3258 and 164.1432. Since pteriatoxins are thought to result from the shellfish metabolism of pinnatoxins, it is also interesting to compare the MS^2^ spectra of the compound identified with the one of pinnatoxins-G detected in the same sample, to be sure. The profile presented strong similarities. In addition to the three specific product ions of the PtTXs, we observed product ions shared with PnTX-G at m/z 554.3837, 440.3151, with a profile distribution comparable in terms of relative abundance. Since the three PtTXs are isomers, it is complex to distinguish them. According to the fragmentation patterns proposed by Takada et al. [7], most of the product ions at m/z 164, 177, 204, 220, 230, 258, 320, 342, 432, 458, 542, 572, 586, 744 and 787 are shared between the three toxins. The product ions at m/z 668 and 694 were only reported for PtTX-A, whereas the product ions at m/z 710 and 712 seem to be specific to the PtTX-B and PtTX-C stereoisomers. Based on this study, the LC-MS/HRMS data obtained did not allow us to identify an individual analog. 

Furthermore, PtTX-A, PtTX-B and PtTX-C, were also screened by LC-MS/MS through selected reaction monitoring (SRM) scans. As a first step, the precursor ion at m/z 831.5, corresponding to the m/z ratio of the three protonated PtTXs [M + H]^+^, was selected, and the product ions at m/z 164.2 and 458.3 were monitored. Chromatographic peaks were observed in all samples at a retention time of 4.0 min for both transitions. The compound eluted before PnTX-E, -F, -G and -H. Indirect quantification with the PnTX-G calibration curve was carried out. For the samples collected in December, levels were comprised between the estimated limit of detection (LOD; 0.1 µg/kg) and the limit of quantification (LOQ; 0.3 µg/kg). For the other months, trace levels ranged between 0.3 and 0.5 µg/kg, with the maximum value observed in August. The ion ratio m/z 458.3/164.2 calculated from this sample was 56.8%.

**Figure 5:**
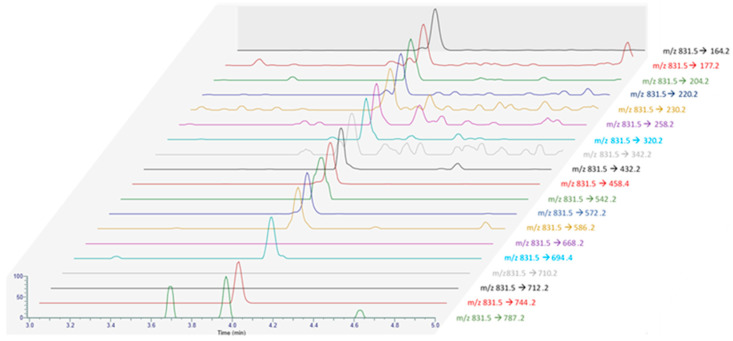
MRM acquisition obtained from the extract of the June sample (18 BM 161) with all the product ions reported by Takada et al. [7].

To try to identify additional product ions compared to LC-MS/HRMS data, SRM scans targeting the precursor ion at m/z 831.5, and the 19 PtTXs’ product ions reported by Takada et al. [7] were monitored from the August sample with instrumental method B.3 (Figure 5) [7]. Interestingly, a peak was observed at the same retention time for all the products ions shared between the three analogs (3.97 ± 0.01 min). No peaks were detected for the chromatographic traces corresponding to the product ions at m/z 710 and 712, which seems characteristic of PtTX-B and PtTX-C stereoisomers. For PtTX-A, the product ions at m/z ratio of 694 were detected; however, no peak was observed for the specific product ion at m/z 668. As this compound was detected at a trace level, it is possible that the signal for several product ions which cannot be detected are below the detection limit. 

Considering all the information collected, this compound was attributed putatively to PtTXs, without ensuring the identify of a specific analog. Molecular mass matching with PtTXs was clearly observed, and we noted strong similarities with PtTX-A (16/17 product ions observed). If this compound is not a known PtTX, it could be a new analog of PtTXs/PnTXs. 

### 2.3. Portimines and Kabirimine

Portimine-A was detected by LC-HRMS through full MS/dd-MS^2^ acquisition mode in samples 18 BM 122, 18 BM 150 and 18 BM 161. All the identification criteria described in the materials and methods section were fulfilled. The retention time was 5.31 min and the following product ions were systematically observed through the dd-MS^2^ acquisitions: 134.05998, 148.07550, 246.14851, 356.22186 [M + H − H_2_O − CO]^+^, 366.20587 [M + H − 2H_2_O]^+^ and 384.21634 [M + H − H_2_O]^+^. A peak was also observed at the correct retention time for sample 18 BM 107, which had a satisfactory isotopic pattern with a mass error ≤±5 ppm. Nevertheless, the signal was not sufficiently high to trigger an MS^2^ acquisition. LC-MS/MS analysis with instrumental method B.1 allowed for the detection of portimine-A throughout 2018, due to higher sensitivity (Table 1). The maximum level was observed in June (75.6 µg/kg). For each sample analyzed, the total toxin level measured was higher than the free toxin level. Considering expanded uncertainty (k = 2) of 44% estimated from the expected reproducibility assessed by the Horwitz function revised by Thompson [48], no uncertainty overlap was observed for samples 18 BM 012, 18 BM 026, 18 BM 065, 18 BM 150, 18 BM 194 and 18 BM 257, suggesting the potential presence of unknown portimine-A esters. Of note, the biggest difference between the total and free toxin was observed for the July sample (18 BM 150), with a difference of 27 µg/kg. 

**Figure 6:**
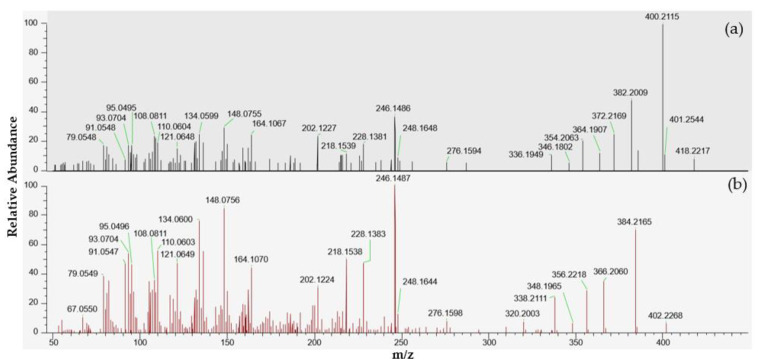
LC-MS/HRMS spectra obtained from the July sample (18 BM 150). (**a**) High-resolution mass spectrum obtained after fragmentation of the precursor ion at m/z 418.2220 corresponding to an elemental composition C_23_H_31_NO_6_, similar to portimine-B. (**b**) High-resolution mass spectrum of portimine-A.

A small peak at 4.78 min corresponding to the [M + H]^+^ protonated molecule of portimine-B (C_23_H_32_NO_6_) was observed in the extracts of samples 18 BM 122 and 18 BM 150. As for portimine-A, no [M + NH4]^+^, [M + Na]^+^ or [M + K]^+^ adducts were detected. These samples were collected in June and July. Since the levels measured were low, both samples were concentrated twenty times through SPE cleanup to obtain an enriched spectrum, and tSIM/dd-MS^2^ acquisitions were carried out, as for PtTXs (instrumental method A.2). Thanks to this strategy, more comprehensive high-resolution MS^2^ spectra were obtained, in particular for the July sample (Figure 6). The elemental compositions of the principal product ions are presented in Table 2. In addition to the precursor ion at m/z 418.2217, we observed product ions at m/z 400.2115 [M + H − H_2_O]^+^, 382.2009 [M + H − 2H_2_O]^+^, 372.2169 [M + H − H_2_O − CO]^+^, 364.1907 [M + H − 3H_2_O]^+^, 354.2063 [M + H − 2H_2_O − CO]^+^, 346.1802 [M + H − 4H_2_O]^+^ and 336.1949 [M + H − 3H_2_O − CO]^+^. All these product ions are also observed in the MS^2^ spectrum of portimine-B reported by Fribley et al. [20], for m/z acquired between 270 and 420. In the literature, no MS^2^ information was available for m/z ratios lower than m/z 270, nevertheless we observed strong similarities with the MS^2^ spectrum of portimine-A (Figure 6). In the m/z range of 79 to 248, the compound identified shared no less than 136 product ions with portimine-A, with m/z differences systematically ≤2 ppm.

In parallel, LC-MS/MS analysis through product ion scan acquisition mode were also carried out to make use of the higher sensitivity of the instrument used. A peak was observed at 2.9 min, and its intensity was two times higher in sample 18 BM 150 compared to sample 18 BM 122. The MS^2^ spectra obtained after selecting the precursor ion at m/z 418.2 also presented numerous similarities with the MS^2^ spectrum reported by Fribley et al. for m/z ranging between 270 and 420 [20]. As with the HRMS acquisition, the principal product ions were all observed at m/z 400.2 [M + H − H_2_O]^+^, 382.2 [M + H − 2H_2_O]^+^, 372.2 [M + H − H_2_O − CO]^+^, 364.2 [M + H − 3H_2_O]^+^, 354.2 [M + H − 2H_2_O − CO]^+^, 346.1 [M + H − 4H_2_O]^+^ and 336.1 [M + H − 3H_2_O − CO]^+^ (Figure S2). In addition, the product ion observed at m/z 284.1255 in the original spectrum of portimine-B, was also observed at m/z 284.0, but with lower intensity. As for the spectrum obtained through HRMS, we observed a series of product ions shared with portimine-A for m/z below 270: m/z 95.1, 110.0, 121.1, 134.0, 148.0, 202.1, 218.1, 228.1 and 246.1. 

Considering all the information gathered, this compound is clearly correlated to portimines, and was attributed to portimine-B with an identification confidence level of 2a, according to the classification proposed by Schymanski et al. [52]. If a reference material had been available, knowledge of the retention time of portimine-B could have enabled us to confirm the identification of this toxin, and rule out the possibility of the discovery of an unknown isomer of portimine-B.

For kabirimine, no features matched in any mussel samples.

## 3. Discussion

The present work first studied the occurrence of *V. rugosum* toxins and their metabolism products in shellfish from Ingril Lagoon, France. The high sensitivity of triple-quadripole LC-MS/MS instruments and the high-resolution power of LC-MS/HRMS demonstrated their utility to investigate the toxinic profile of the mussel samples in-depth. The pinnatoxin profile observed in mussels was similar to those already observed at this locality between 2013 and 2017 [22,38,39,53]. PnTX-G is the main analog produced, followed to a lesser extent by PnTX-A. Except for December, PnTX-G levels consistently exceeded the limit concentration of 23 µg/kg, which is expected to result in adverse effects in humans. It is worth noting that in 2020, competent authorities decided to ban the production and collection of shellfish in this lagoon due to the regular non-compliance of PnTX-G levels measured with this limit. The maximum value observed in the present work was 467.5 µg/kg in July. This value is relatively low compared to the maximum value observed previously in this lagoon, a well-known hotspot for *V. rugosum* growth. Between 2010 and 2017, the maximum values ranged between 568 and 1244 µg/kg with high levels mostly observed between June and September [22,39,53]. This seasonal variability is similar to that observed in the present work since an increase in PnTX-G levels was observed between June and October 2018. Within the national observation and monitoring program for phytoplankton and hydrology in coastal waters, measurements of water temperatures indicated a range between 10 and 13 °C from January to May on this site [54]. The increase in PnTX-G levels in mussels corresponded to the moment when the temperature of the lagoon increased quickly from 11.9 (May) to 21.4 °C (June). The temperature then stayed at >21 °C until September, with values comprised between 21.4 and 24.5 °C (August) and then started to decrease constantly from October (18.3 °C) to December (11.5 °C). *V. rugosum* has been described as euryhaline and thermophile, which could explain why this dinoflagellate develops in situ only from June to September [55]. Laboratory experiments have shown that *V. rugosum* can grow at temperatures ranging from 20 to 30 °C, with an optimal temperature of 25 °C. For salinity, the optimal conditions are comprised between 20 and 40 g/L. Throughout 2018, salinity remained within this window, with values ranging between 20.8 and 39.3 g/L [54]. 

PnTX-B, PnTX-C, PnTX-E, PnTX-F and PnTX-H were not detected. To date, none of these analogs have been identified in Europe, but they have been reported in Australia, Japan and New-Zealand.

A total of 13 fatty acid esters of PnTX-G were identified among the 26 analogs discovered by Mc Carron et al. [17]. In the present work, we did not observe a significant increase in PnTX-G concentrations after hydrolysis treatment. This is certainly due to the trace levels of PnTX-G esters observed in the mussel samples. These results are consistent with those in the study carried out by Araoz et al. [19], which enabled the detection of trace levels of 28-*O*-palmitoyl ester of PnTX-G in digestive glands of mussels from the same lagoon. The other 12 esters of PnTX-G identified in the present work were not previously detected elsewhere, other than in Canada. Interestingly, four of the fatty acids involved in PnTX-G conjugation correspond to the dominant fatty acids observed in a mussel composition study of the species *Mytilus galloprovincialis* from the Black Sea [56]. In fact, C16:0, C22:6, C20:5 and C16:1 were the most commonly observed among the 27 fatty acids screened. All the fatty acids conjugated to PnTX-G are reported in the composition of mussels. The knowledge of esterification metabolism of marine biotoxins in shellfish is still limited. For okadaic acid, dinophysistoxin-1, dinophysistoxin-2 and gymnodimine-A, the binding capacity of fatty acids is species-specific, which supports the hypothesis that esterification is catalyzed by certain enzymes, in particular acetyl-coenzyme A carboxylase, fatty acid synthase, lipoprotein lipase and hepatic lipase [57,58]. For okadaic acid, most of the toxins conjugated with fatty acids were found in the feces of bivalves, suggesting that the metabolic transformation of okadaic acid and dinophysistoxin-1 to acyl esters was a key step in the depuration process of mussels via egestion of feces [59].

Portimine-A was also detected throughout 2018, with maximum values observed between June and July. As for PnTX-G, this period seems to be favorable for the production and accumulation of portimine-A in mussels. The lower levels of portimine-A in mussels compared to PnTX-G levels were also observed previously, after contamination/decontamination experiments with mussels exposed to *V. rugosum* cells [24]. According to the authors, a lower capacity for accumulation or biotransformation processes could explain these results.

High-resolution mass spectrometry enabled the identification of two additional compounds suspected to be toxins. The first one was considered to be an analog of PtTXs, but in the absence of any reference material, and with no additional structural information it was not possible to identify the specific PtTX analog, since the three analogs are isomers. Therefore, this compound was putatively attributed to PtTXs with an identification confidence level of 3 according to the confidence levels proposed by Schymanski et al. [52]. It was detected throughout 2018, at low levels (≤0.5 µg/kg; indirectly quantified from PnTX-G calibration curve), with a maximum observed in August. It should be noted that since their isolation and identification in 2001, PtTXs have only been detected in Okinawan bivalve *Pteria penguin* [7,10]. The second compound had the same elemental composition as portimine-B (C_23_H_32_NO_6_), and was identified in July sample. The MS^2^ spectrum obtained presented numerous similarities with the spectra of both portimine-B published by Fribley et al. [20], and portimine-A detected in the same sample. The availability of a reference material would have allowed us to confirm its identity, in particular by checking retention time matching, to be certain that this is not an unknown analog of portimine, constitutional isomer of portimine-B. Therefore, this compound was attributed to portimine-B with an identification confidence level of 2a (probable structure). To date, the accumulation of this recently discovered toxin in shellfish has never been reported in the world. Finally, we checked for the presence of other compounds with a molecular mass similar to portimine-B and PtTXs in the Comprehensive Marine Natural Products Database (CMNPD) [60]. No matches were found.

## 4. Materials and Methods

### 4.1. Chemicals and Reagents

All solutions were prepared with liquid chromatography-mass spectrometry (LC-MS) grade chemicals and ultrapure water (18.2 MΩ/cm) obtained by purifying distilled water with a Milli-Q system. A PnTX-A reference material, with a concentration of 5.0 µg/mL, was purchased from Abraxis LLC (Warminster, PA, USA). A PnTX-G certified reference material of 1.92 ± 0.09 µg/mL was purchased from NRCC (Halifax, NS, Canada). PnTX-E, PnTX-F, PnTX-H and portimine-A reference material from the Cawthron Institute (Nelson, New Zealand) were also used. For the last three PnTX analogs, three individual stock solutions at 10.0 µg/mL were prepared in methanol from the 10 µg of each toxin. For portimine-A, a stock solution at 11.9 µg/mL was prepared in methanol from the 11.9 µg of toxin. Working solutions were prepared from the stock solutions to spike the blank samples used as quality control, but also to prepare calibration curves composed of at least five levels ranging from the LOQ to 50 ng/mL. Acetonitrile, methanol, acetic acid and formic acid were purchased from Fisher Scientific (Loughborough, UK). Ultra-pure-grade carrier argon (Ar, 99.9999% pure) was purchased from Linde Gas (Montereau-Fault-Yonne, France). Nitrogen was also purchased from Linde Gas (N2, 99.999% pure).

The protocol implemented for the synthesis of 28-*O*-myristoyl PnTX-G (C14:0-PnTX-G) requires the use of myristic anhydride (purity ≥95.0%) obtained from Sigma-Aldrich (Saint-Louis, MO, USA). Basic reagents were also used as catalysts. In addition, 4-(Dimethylamino)pyridine (ReagentPlus, purity ≥99%) and pyridine anhydrous (purity ≥99.75%) were purchased from Sigma-Aldrich.

### 4.2. Sampling Design

Throughout 2018, mussels (*Mytilus galloprovincialis*), which is the predominant bivalve in this geographical area, were sampled from Ingril Lagoon, situated on the Mediterranean coast of France. These samples were collected in parallel to the French program for monitoring emerging toxins (EMERGTOX network). A total of 1 kg of bivalve mollusks was sampled monthly (*n* = 12). 

### 4.3. Sample Preparation

#### 4.3.1. Extraction of Toxins

Mussels were prepared according to the standard operating procedure of the European Union Reference Laboratory for Marine Biotoxins (EURL-MB) for determination of the okadaic acid, pectenotoxin, azaspiracid, and yessotoxin group toxins using LC-MS/MS [61]. In 2010, this protocol was validated in-house at our laboratory and has since been ISO 17025 accredited. A 2.00 ± 0.05 g portion of tissue was homogenized for 2 min with 9 mL of MeOH using a Polytron (Kinematica AG; Luzern, Switzerland) at 10,000 ± 500 rpm. After extraction, the extract was centrifuged at 6000× *g* for 10 min, and the supernatant was collected into a 20 mL volumetric flask. A second extraction with 9 mL of MeOH was carried out for 1 min. Finally, this raw extract volume was adjusted to 20 mL with MeOH, and filtered through a 0.45 µm centrifuge filter (Thermo Fisher Scientific; San Jose, CA, USA) at 2000× *g* for 2 min, prior to direct analysis or hydrolysis of the fatty acid ester forms of PnTX-G. 

#### 4.3.2. Hydrolysis of Fatty Acid Esters of PnTX-G

Fatty acid esters of PnTX-G were hydrolyzed to transform them into PnTX-G (additionally to the free PnTX-G already present). The comparison between the results obtained with and without the hydrolysis step allowed us to estimate the level of fatty acid ester derivatives of PnTX-G. Then, 500 μL of aqueous NaOH 2.5 M was added to 4 mL of methanolic extract in a glass tube. After vortex mixing, the tube was heated to 76 ± 4 °C for 40 min. Once cooled to room temperature, the extract was neutralized with 500 μL of aqueous 2.5M HCl. Finally, the extract was filtered on a 0.20 µm polytetrafluoroethylene (PTFE) syringe filter (Chromafil, Macherey-Nagel GmbH & Co. KG; Düren, Germany) prior to analysis.

#### 4.3.3. Solid Phase Extraction Clean-Up and Concentration 

A solid phase extraction clean-up was implemented to investigate the presence of PtTXs and portimine-B by concentrating the raw extracts of samples 18 BM 122, 18 BM 150 and 18 BM 161. A 60 mg/3 mL Strata-X cartridge (Phenomenex; Torrance, CA, USA) was conditioned with 3 mL MeOH followed by 3 mL 30% MeOH. Seven milliliters of water was added to 3 mL of raw extract to be deposited. The cartridge was washed with 3 mL of a solution containing water/MeOH/NH_4_OH at a ratio of 89:10:1 (v/v/v). The cartridge was dried for 3 min. Toxins were eluted with 3 mL MeOH and collected in a clean glass tube. Before and after elution, the cartridge was dried for 3 min under reduced pressure of 10 kPa. Afterwards, the extract was evaporated to dryness under a gentle stream of N_2_ at 40 °C, and 150 µL MeOH was added.

### 4.4. Analysis by Liquid Chromatography Coupled to Mass Spectrometry 

#### 4.4.1. LC-MS/HRMS Analysis

LC-HRMS analyses were performed for the suspect screening of *V. rugosum* toxins and their metabolites in shellfish, but also for in-depth investigation when reference materials were not available. The LC system was an Ultimate 3000 (Thermo Fisher Scientific). The separation of toxins was performed using a Hypersil Gold (50 × 2.1 mm, 1.9 μm particle sizes, 130 Å) from Thermo Fisher Scientific. The column temperature was set to 40 °C. Eluent A was composed of water and eluent B of acetonitrile/water at a ratio of 95:5 (v:v), with both eluents containing 50 mM formic acid and 2 mM ammonium formate. The LC flow rate was 0.400 mL/min and the separation gradient was programmed as follows: 5% B hold 1.0 min, 5–100% B in 9 min, 100% B hold 9.5 min, 100–5% B in 0.5 min, and 5% B hold 3.0 min. Furthermore, 2 μL was injected into the system.

The detection of toxins was performed with a Q Exactive hybrid quadrupole-orbitrap mass spectrometer (Thermo Fisher Scientific), equipped with an electrospray ionization (ESI) source (HESI-II probe). The spray voltage was 3500 V in positive ionization mode. The source temperature was set at 425 °C and the capillary temperature at 263 °C. Nitrogen was used as the nebulizing gas with a sheath gas flow of 50 (arbitrary units), an auxiliary gas flow of 13 (arbitrary units) and a sweep gas flow of 3 (arbitrary units). The S-lens RF level voltage was set to 55 V. The collision gas was nitrogen. For suspect screening analysis with instrumental method A.1, acquisitions were performed using full MS data-dependent MS^2^ mode (full MS/dd-MS^2^). For the full MS acquisition, a resolution of 70,000 full width at half maximum (FWHM), an AGC target of 3 × 10^6^, a max IT of 100 ms and a scan range of 100–1100 were defined. For dd-MS^2^ acquisition, the following parameter values were selected: resolution 17,500 FWHM, isolation window 1.5 m/z, AGC target 5 × 10^4^ (min AGC target of 8 × 10^2^) and max IT 50 ms. A top N of 5 was retained with an intensity threshold of 1.6 × 10^4^, and a dynamic exclusion of 10 s. Two normalized collision energies were selected: 37 and 47. Several minimum criteria were defined for toxin identification. A mass error ≤±5 ppm for precursor ion was required and the isotopic pattern of, at least, the M, M+1 and M+2 isotopes should match with the theoretical profile in terms of intensity (<25%) and mass error (≤±10 ppm). An MS^2^ spectrum should be acquired with the data-dependent mode carried out with at least three product ions with a mass error <±10 ppm. When a standard was available, the retention time (RT) should be within ±0.1 min. LOD was defined as the lowest level of the calibration curve fulfilling the identification criteria. Based on this approach, the LODs of PnTX-A, PnTX-G and portimine-A were estimated at 10 µg/kg, 2 µg/kg and 4 µg/kg, respectively. 

Investigations of the compounds suspected to be PtTXs and portimine B were carried out through targeted-selected ion monitoring with data-dependent MS^2^ scans (t-SIM/dd-MS^2^), and parallel reaction monitoring (PRM). For t-SIM/dd-MS^2^ acquisitions (instrumental method A.2), the following SIM parameter values were selected: resolution of 70,000 FWHM, AGC target of 5 × 10^4^, max IT of 100 ms and a scan range of 150–2000; and for dd-MS^2^: resolution 35,000 FWHM, isolation window 2.0 m/z, AGC target 2 × 10^5^ (min AGC target of 8 × 10^3^) and max IT 100 ms. A top N of 10 was retained with an intensity threshold of 8 × 10^4^. For PRM acquisitions (instrumental method A.3), an MS^2^ resolution of 70,000 FWHM, an AGC target of 2 × 10^5^, a max IT of 240 ms, an isolation window of 2.0 m/z and max IT of 240 ms were defined. For both methods, normalized collisions of 35 and 42 were selected for PtTXs and portimine-B, respectively.

Instrument control was handled by a computer equipped with Thermo Q Exactive version 2.9 Build 2926, Xcalibur version 4.1.50 and TraceFinder version 4.1—EFS (Thermo Fisher Scientific). Acquired data were processed with Freestyle version 1.8.51.0 and Compound Discoverer 3.30.550 (Thermo Fisher Scientific). 

#### 4.4.2. LC-MS/MS Analysis

LC-MS/MS analyses were implemented for qualitative and quantitative analyses of *V. rugosum* toxins and their metabolites in shellfish with several instrumental methods. The LC system was a Vanquish Horizon (Thermo Fisher Scientific). The separation of toxins was performed using the same LC column, column temperature, eluents and LC flow rate as for LC-HRMS analysis. The detection of toxins was performed with a TSQ Altis triple quadrupole mass spectrometer (Thermo Fisher Scientific), equipped with an electrospray ionization (ESI) source (Opta-Max NG). The mass spectrometer was operated in selected reaction monitoring (SRM) mode. The spray voltage was 500 V in positive ionization mode. The source temperature was set at 350 °C and capillary temperature at 325 °C. Nitrogen was used as the nebulizing gas with a sheath gas pressure of 37 (arbitrary units), an auxiliary gas pressure of 6 (arbitrary units) and a sweep gas pressure of 2.9. The collision gas was argon, with a gas pressure of 1.5 mTorr. Instrumental method B.1 was implemented for the quantitative analysis of *V. rugosum* toxins and their metabolites in shellfish, except the esters of PnTX-G that were screened qualitatively with a specific method (instrumental method B.2). Even though no reference material was available for portimine-B, this recently discovered toxin was included in the method based on the MS^2^ spectra provided by Fribley et al. [20], but also after implementation of product ion scans from the samples studied in the present work. Kabirimine could not be included since we had neither a standard for method optimization (commercially unavailable), nor an MS^2^ spectrum for this metabolite. One transition was used for quantification (Q) and another as a qualifier transition (q). The optimized compound-dependent parameters are listed in Table 3. For portimines, the RF lens voltage was set to 70 V, whereas for PnTXs and PtTXs a value of 105 V was retained. A mass resolution of 0.7 Da (full width at half maximum) was set for the first and the third quadrupoles (Q1 and Q3). When reference materials were available, recoveries were assessed at a level of 100 µg/kg. For PnTX-A, PnTX-E, PnTX-F, PnTX-G and PnTX-H, the values ranged between 82 and 102% for the non-hydrolyzed extracts, whereas for portimine-A, recovery was 84%. After hydrolysis treatment, recoveries of PnTX-A, PnTX-G and portimine-A were 82%, 83% and 84%, respectively. LOQ was defined as the lowest level of the calibration curve with a signal-to-noise ratio higher than 10 for the quantitative and qualifier transition. LOQ was divided by three to establish the LOD. Based on this approach, the LOD of PnTX-A, PnTX-G, PnTX-H and portimine-A was estimated to be 0.13 µg/kg, and the LOQ 0.4 µg/kg. For PnTX-E and PnTX-F, 0.67 and 2.0 µg/kg were achieved, respectively.

Instrumental method B.2 was implemented for LC-MS/MS targeted analysis of the fatty acid esters of PnTX-G. In addition to PnTX-G, the 26 fatty acid conjugates of PnTX-G reported by McCarron et al. [17] were monitored through SRM acquisitions (Table S1). Two specific product ions were chosen for each analog with m/z ratios of 164.21 and 458.38, and a respective CE of 54 and 44 V.

Instrumental method B.3 was implemented for the identification of PtTXs through SRM scans. The protonated molecules at m/z 831.5 [M + H]^+^, and the 19 PtTXs products ions reported by Takada et al. [7] were selected. Depending on the product ions, CE were comprised between 40 and 65 V.

For the entire LC-MS/MS method, instrument control and acquired data were handled by a computer equipped with TSQ Altis Tune Application version 3.2.2625.33, Xcalibur version 4.2.47, TraceFinder version 4.1—EFS and Freestyle version 1.8.51.0 (Thermo Fisher Scientific).

### 4.5. Synthesis of Fatty Acid Esters of Pinnatoxin-G

The fatty acid anhydride reaction route was chosen for the acylation of PnTX-G, and the formation of 28-O-myristoyl PnTX-G. Pyridine and DMAP were used for the activation of the fatty acid anhydride. The molar ratio toxin/reagents was ≥1:10,000. Before use, all the glassware used was kept at 150 °C in a laboratory oven for at least 2 h to remove any remaining moisture. Afterwards, glassware was cooled, and kept in a desiccator. An aliquot of 0.25 µg of toxin was taken from the standard and evaporated to dryness under an N_2_ stream. Then, the tube was placed in a desiccator for at least 2 h. The dry residue was dissolved with 1.0 mL of 100 mM myristic anhydride, and 100 mM DMAP in anhydrous pyridine. After vortex-mixing, solutions were reacted either by using a Reacti-Therm (Thermo Fisher Scientific) at a controlled temperature of 75 °C for 30 min, or at room temperature. Once the reaction time was completed, the solutions were cooled and evaporated to dryness under an N_2_ stream at 50 °C by the combined use of a Reacti-Therm system and a Reacti-Vap module (Thermo Fisher Scientific). Finally, the dry residues were dissolved in 4 mL MeOH, vortex mixed and filtered on a 0.20 µm PTFE syringe filter (Chromafil, Macherey-Nagel GmbH & Co. KG) for LC-MS/MS analysis. Based on peak intensity, and assuming equal instrumental responses, both temperature conditions enabled preparation of semi-synthesized products with a conversion of PnTX-G into 28-O-myristoyl PnTX-G > 99.9%.

## 5. Conclusions

Pinnatoxins, pteriatoxins, portimines and kabirimine were monitored monthly in mussels from Ingril Lagoon, France, a well-known hotspot for *V. rugosum* growth. This short time-series study gave an overview of the presence and seasonal variability of these toxins throughout 2018. Among the algal toxins screened, PnTX-A, PnTX-G and portimine-A were identified and quantified with maximum values observed in June and July, when water temperature increased. Except for the December sample, PnTX-G levels consistently exceeded the concentration limit of 23 µg/kg, which is expected to result in adverse effects in humans. In addition, two other compounds were detected. The first one was putatively attributed to an analog of PtTXs: PtTX-A, PtTX-B or PtTX–C. These three products of shellfish metabolism have so far only been reported in Japan. More information was gathered for the second one, considered to be portimine-B after in-depth mass spectrometry investigations. The accumulation of this recently discovered toxin has never been reported in the world. In both cases, the lack of standards did not enable definitive identification but there is nevertheless sufficient evidence indicating that these compounds are analogs of PnTXs/PtTXs and portimines, sharing the same molecular formula as PtTXs and portimine-B. This study also demonstrated, for the first time, the presence of a series of PnTX-G fatty acid esters at low levels in European shellfish. As with other marine biotoxin fatty acid conjugates, these products of shellfish metabolism are probably hydrolyzed by lipases and other enzymes into the gastrointestinal tract during human digestion to release PnTX-G whose acute toxicity has been demonstrated. Therefore, in addition to the obtention of more toxicity data, supplementary contamination data for *V. rugosum* toxins and their biotransformation products are required. Our results contribute to a better hazard characterization to establish whether exposure to these metabolites presents a risk for humans, and highlight the need for further studies targeting larger geographical areas and different shellfish species.

## Figures and Tables

**Table 1:** Quantification of PnTX-A, PnTX-G and portimine-A in mussels collected from Ingril Lagoon in 2018.

Sample	Sampling Month	Levels (µg/kg)					
		PnTX-A		PnTX-G		Portimine-A	
		Free	Total	Free	Total	Free	Total
18 BM 012	January	4.2	5.9	56.7	55.4	0.7	1.9
18 BM 026	February	4.6	6.3	68.8	68.2	0.6	1.0
18 BM 042	March	2.0	2.8	38.4	37.6	<LOQ *	0.7
18 BM 065	April	2.1	2.8	59.3	59.3	0.4	0.7
18 BM 107	May	1.1	2.1	48.1	52.0	2.4	3.0
18 BM 122	June	4.0	4.5	230.0	235.5	75.6	78.7
18 BM 150	July	6.7	6.2	467.5	475.2	32.4	59.4
18 BM 161	August	6.3	10.1	367.3	390.5	8.9	10.2
18 BM 194	September	3.1	3.9	319.4	314.4	0.9	1.7
18 BM 257	October	4.6	4.6	321.6	316.9	0.5	0.8
18 BM 271	November	1.2	2.1	27.4	25.5	<LOQ *	0.6
18 BM 286	December	2.3	3.1	12.7	14.3	0.5	0.6

* < LOQ: comprised between the limit of detection (0.13 µg/kg) and the limit of quantification (0.4 µg/kg).

**Table 2:** Fragmentation of the precursor ion at m/z 418.2217 for the July sample (18 BM 150), and elemental composition of the products ions.

m/z	Elemental Composition	Δ ppm
79.0548	No proposal	/
91.0547	No proposal	/
93.0704	No proposal	/
95.0495	C_6_H_7_O	3.8
108.0811	C_7_H_10_N	3.0
110.0604	C_6_H_8_NO	3.2
121.0648	C_8_H_9_O	0.4
134.0755	C_8_H_8_NO	−1.2
148.0755	C_9_H_10_NO	−1.3
164.1067	C_10_H_14_NO	−1.6
202.1224	C_13_H_16_NO	0.4
218.1539	C_14_H_20_NO	−0.4
228.1381	C_15_H_18_NO	−0.9
246.1486	C_15_H_20_NO_2_	−1.0
336.1949	C_22_H_26_NO_2_; [M + H − 3H_2_O − CO]^+^	−2.6
346.1802	C_23_H_24_NO_2_; [M + H − 4H_2_O]^+^	0.1
354.2063	C_22_H_28_NO_3_; [M + H − 2H_2_O − CO]^+^	−0.1
364.1907	C_23_H_26_NO_3_; [M + H − 3H_2_O]^+^	−0.1
372.2169	C_22_H_30_NO_4_; [M + H − H_2_O − CO]^+^	0.0
382.2009	C_23_H_28_NO_4_; [M + H − 2H_2_O]^+^	−0.9
400.2115	C_23_H_30_NO_5_; [M + H − H_2_O]^+^	−0.8
418.2217	C_23_H_32_NO_6;_ [M + H]^+^	−1.6

**Table 3:** Compound-dependent tandem mass spectrometry parameters for instrumental method B.1.

Compound	Precursor Ion (m/z)	Product Ion * (m/z)	Collision Energy (V)
PnTX-A	712.5	164.2 (Q)	77
		458.3 (q)	61
PnTX-B/C	741.4	164.2 (Q)	77
		458.3 (q)	61
PnTX-D	782.4	164.2 (Q)	77
		488.3 (q)	61
PnTX-E	784.4	164.2 (Q)	77
		488.3 (q)	61
PnTX-F	766.4	164.2 (Q)	77
		488.3 (q)	61
PnTX-G	694.5	164.2 (Q)	77
		458.3 (q)	61
PnTX-H	708.5	164.2 (Q)	77
		488.3 (q)	61
PtTXs	831.5	164.2 (Q)	77
		458.3 (q)	61
Portimine-A	402.2	384.2 (Q)	22
		246.2 (q)	28
Portimine-B	418.2	400.2 (Q)	22
		246.2 (q)	28

* Q: Quantitative transition; q: qualifier transition.

## Data Availability

The original data presented in the study are included in the article/Supplementary Material; further inquiries can be directed to the corresponding author.

## Supplementary Materials

The following supporting information can be downloaded at: https://www.mdpi.com/article/10.3390/md21080429/s1, Figure S1. Detection of PnTX-G and 13 of its fatty acid esters by LC-MS/MS in mussels collected in June (sample 18 BM 122). Chomatogram created with R version 4.0.3, and with the Plotly package version 4.10.0 [51,53]; Figure S2. Product ion scan obtained from the precursor ion at m/z 418.0 (same as the protonated portimine-B [M + H]^+^) for the compound eluted at 2.9 min in sample 18 BM 150; Table S1: Molecular formula and monoisotopic mass of the protonated PnTX-G esters [M + H]^+^ reported by McCarron et al.
