# Supplementary material for: Exploration of Vulcanodinium rugosum Toxins and their Metabolism Products in Mussels from the Ingril Lagoon Hotspot in France
Source: Mar Drugs. 2023 Jul 29;21(8):429. doi: 10.3390/md21080429 (PMC10455521; doi:10.3390/md21080429)
Supplement: Supplementary file 1 [file marinedrugs-21-00429-s001.zip › marinedrugs-2521741-SM.pdf]

marine drugs

Supplementary Materials

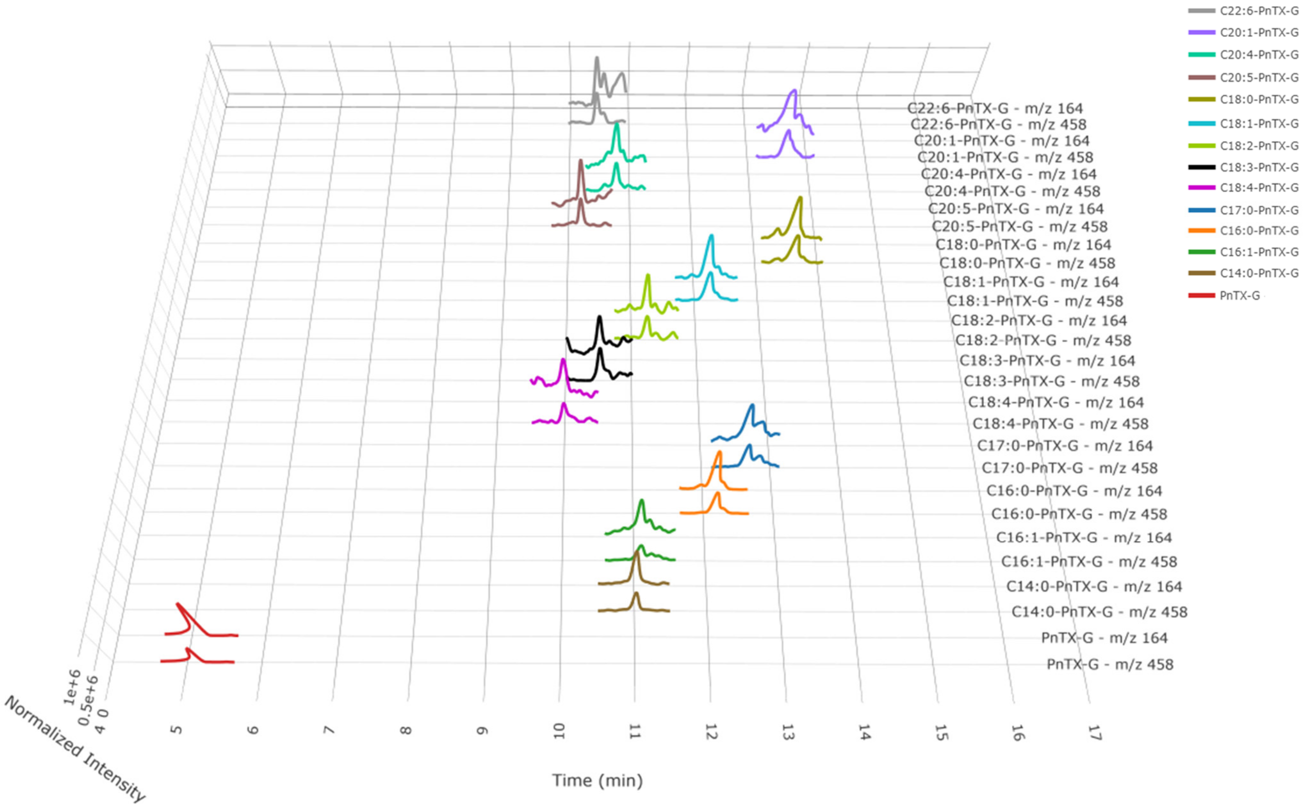

Figure S1. Detection of PnTX-G and 13 of its fatty acid esters by LC-MS/MS in the mussel sample collected in June (18 BM 122). Chromatogram created with R version 4.0.3, and with the Plotly package version 4.10.0 [49,51].

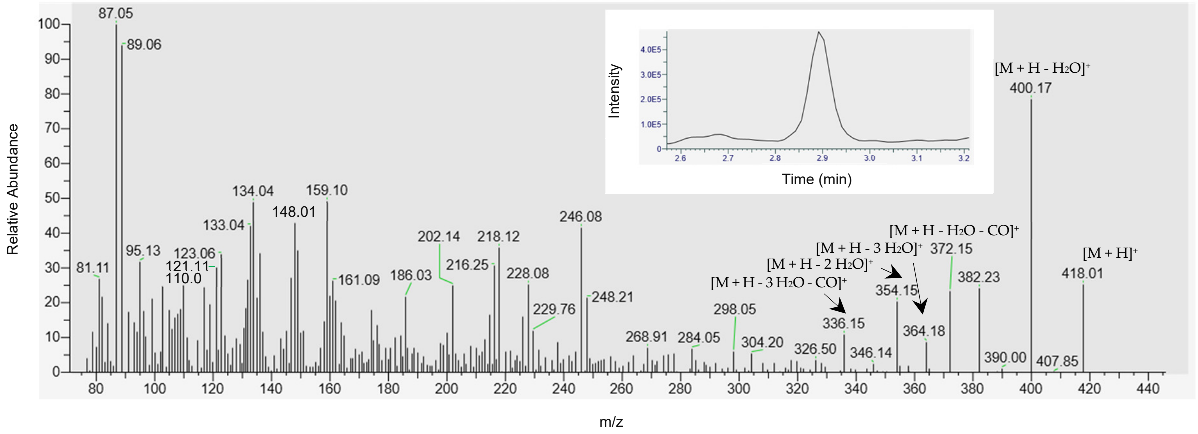

Figure S2. Product ion scan obtained from the precursor ion at m/z 418.2 (same as the protonated portimine-B [M + H]$^+$) for the compound eluted at 2.9 min in sample 18 BM 150.

Table S1. Molecular formula and monoisotopic mass of the protonated PnTX-G esters $[M + H]^+$ reported by McCarron et al. [17].

Fatty acid chain	Systematic name (Trivial name)	Ester molecular formula	Monoisotopic mass of the protonated molecule $[M + H]^+$
C14:0	Tetradecanoic (Myristic)	$C_{56}H_{89}NO_8$	904.66664
C15:0	Pentadecanoic (Pentadecylic)	$C_{57}H_{91}NO_8$	918.68229
C16:2	Hexadecadienoic	$C_{58}H_{89}NO_8$	928.66664
C16:1	Hexadecaenoic	$C_{58}H_{91}NO_8$	930.68229
C16:0	Hexadecanoic (Palmitic)	$C_{58}H_{93}NO_8$	932.69794
C17:2	Heptadecadienoic	$C_{59}H_{91}NO_8$	942.68229
C17:1	Heptadecaenoic	$C_{59}H_{93}NO_8$	944.69794
C17:0	Heptadecanoic (Margaric)	$C_{59}H_{95}NO_8$	946.71359
C18:4	Octadecatetraenoic	$C_{60}H_{89}NO_8$	952.66664
C18:3	Octadecatrienoic	$C_{60}H_{91}NO_8$	954.68229
C18:2	Octadecadienoic	$C_{60}H_{93}NO_8$	956.69794
C18:1	Octadecaenoic	$C_{60}H_{95}NO_8$	958.71359
C18:0	Octadecanoic (Stearic)	$C_{60}H_{97}NO_8$	960.72924
C19:0	Nonadecanoic (Nonadecylic)	$C_{61}H_{99}NO_8$	974.74489
C20:5	Eicosapentaenoic	$C_{62}H_{91}NO_8$	978.68229
C20:4	Eicosatetraenoic	$C_{62}H_{93}NO_8$	980.69794
C20:3	Eicosatrienoic	$C_{62}H_{95}NO_8$	982.71359
C20:2	Eicosadienoic	$C_{62}H_{97}NO_8$	984.72924
C20:1	Eicosenoic	$C_{62}H_{99}NO_8$	986.74489
C22:6	Docosahexaenoic	$C_{64}H_{93}NO_8$	1004.69794
C22:5	Docosapentaenoic	$C_{64}H_{95}NO_8$	1006.71359
C22:4	Docosatetraenoic	$C_{64}H_{97}NO_8$	1008.72924
C22:3	Docosatrienoic	$C_{64}H_{99}NO_8$	1010.74489
C22:2	Docosadienoic	$C_{64}H_{101}NO_8$	1012.76054
C24:6	Tetracosahexaenoic	$C_{66}H_{97}NO_8$	1032.72924
C24:5	Tetracosapentaenoic	$C_{66}H_{99}NO_8$	1034.74489
